# In vivo testing of the low-flow CO_2_ removal application of a compact, platform respiratory device

**DOI:** 10.1186/s40635-020-00329-9

**Published:** 2020-08-17

**Authors:** Alexandra G. May, Ryan A. Orizondo, Brian J. Frankowski, Sang-Ho Ye, Ergin Kocyildirim, William R. Wagner, Jonathan D’Cunha, William J. Federspiel

**Affiliations:** 1 Department of Chemical and Petroleum Engineering, University of Pittsburgh, Pittsburgh, USA; 2 McGowan Institute for Regenerative Medicine, University of Pittsburgh, 3025 East Carson Street, Pittsburgh, PA 15203 USA; 3 Department of Medicine, University of Pittsburgh, Pittsburgh, USA; 4 Department of Surgery, University of Pittsburgh, Pittsburgh, USA; 5 Department of Cardiothoracic Surgery, Children’s Hospital of Pittsburgh, Pittsburgh, USA; 6 Department of Bioengineering, University of Pittsburgh, Pittsburgh, USA; 7 Division of Lung Transplantation/Lung Failure, Department of Cardiothoracic Surgery, University of Pittsburgh Medical Center, Pittsburgh, USA; 8 Department of Critical Care Medicine, University of Pittsburgh Medical Center, Pittsburgh, USA; 9 Clinical and Translational Science Institute, University of Pittsburgh, Pittsburgh, USA

**Keywords:** Carbon dioxide, Hypercapnia, Extracorporeal CO_2_ removal

## Abstract

**Background:**

Non-invasive and lung-protective ventilation techniques may improve outcomes for patients with an acute exacerbation of chronic obstructive pulmonary disease or moderate acute respiratory distress syndrome by reducing airway pressures. These less invasive techniques can fail due to hypercapnia and require transitioning patients to invasive mechanical ventilation. Extracorporeal CO_2_ removal devices remove CO_2_ independent of the lungs thereby controlling the hypercapnia and permitting non-invasive or lung-protective ventilation techniques. We are developing the Modular Extracorporeal Lung Assist System as a platform technology capable of providing three levels of respiratory assist: adult and pediatric full respiratory support and adult low-flow CO_2_ removal. The objective of this study was to evaluate the in vivo performance of our device to achieve low-flow CO_2_ removal.

**Methods:**

The Modular Extracorporeal Lung Assist System was connected to 6 healthy sheep via a 15.5 Fr dual-lumen catheter placed in the external jugular vein. The animals were recovered and tethered within a pen while supported by the device for 7 days. The pump speed was set to achieve a targeted blood flow of 500 mL/min. The extracorporeal CO_2_ removal rate was measured daily at a sweep gas independent regime. Hematological parameters were measured pre-operatively and regularly throughout the study. Histopathological samples of the end organs were taken at the end of each study.

**Results:**

All animals survived the surgery and generally tolerated the device well. One animal required early termination due to a pulmonary embolism. Intra-device thrombus formation occurred in a single animal due to improper anticoagulation. The average CO_2_ removal rate (normalized to an inlet pCO_2_ of 45 mmHg) was 75.6 ± 4.7 mL/min and did not significantly change over the course of the study (*p* > 0.05). No signs of consistent hemolysis or end organ damage were observed.

**Conclusion:**

These in vivo results indicate positive performance of the Modular Extracorporeal Lung Assist System as a low-flow CO_2_ removal device.

## Background

Mechanical ventilation (MV) is currently being used to normalize blood gas tensions in patients with acute respiratory distress syndrome (ARDS) or acute exacerbations of chronic obstructive pulmonary disease (ae-COPD). MV can further injure the lungs via ventilator-induced lung injury (VILI) [1, 2]. Reducing the airway pressures through non-invasive ventilation (NIV) or lung-protective ventilation techniques can improve patient outcomes; however, up to 25% of ae-COPD patients will fail NIV due to hypercapnia [3, 4]. The subsequent hypercapnia and acidosis require invasive MV to normalize blood gases [5, 6].

Extracorporeal CO_2_ removal (ECCO_2_R) devices provide a means to resolve the hypercapnia during lung-protective ventilation and in some instances allow extubation or NIV [7–11]. Typical ECCO_2_R duration for ae-COPD patients is 7 days or less [12]. The ongoing VENT-AVOID (NCT03255057) and REST (NCT02654327) randomized clinical trials aim to further expand the clinical evidence of using vv-ECCO_2_R as an alternative to invasive mechanical ventilation or in conjunction with low tidal volume ventilation, respectively.

We are developing the Modular Extracorporeal Lung Assist System (ModELAS) as a multi-functional respiratory assist technology capable of accommodating three levels of respiratory assist. The ModELAS is a highly compact, integrated pump-lung capable of being worn or integrated onto a wheeled console. The device can be configured to provide pediatric or adult complete respiratory assist (oxygenation and CO_2_ removal) or adult low-flow ECCO_2_R. Selection of the cannula and hollow fiber membrane (HFM) bundle size permits configuration for either pediatric or adult respiratory applications. The pediatric ModELAS utilizes a central cannulation and has been evaluated in acute animal studies [13, 14]. The adult complete respiratory assist application uses a dual lumen cannulation and is currently being evaluated in 30-day animal studies [15, 16].

In the present study, we evaluated the 7-day in vivo performance of the adult low-flow ECCO_2_R ModELAS in an awake ovine model. This is the first in vivo evaluation of the ModELAS configured for adult low-flow ECCO_2_R. This series of animal studies aims to validate benchtop CO_2_ removal and pump performance as well as evaluate sustained device CO_2_ removal, thrombogenicity of the device, and hemocompatibility.

## Methods

**Fig. 1:**
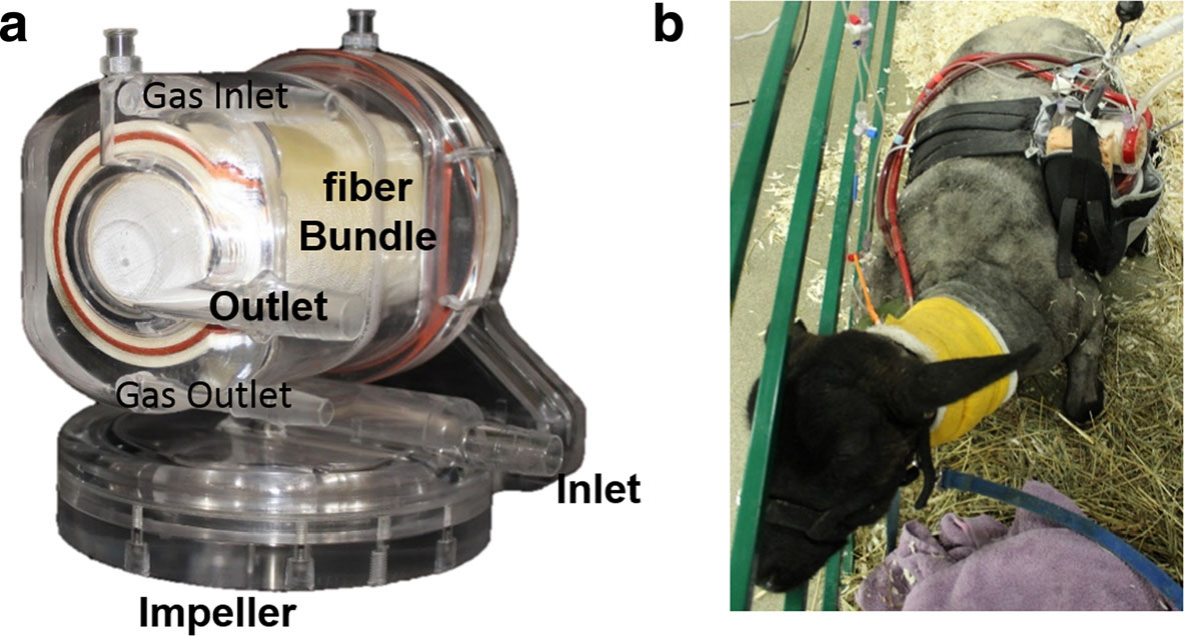
Prototype of the adult low-flow ECCO_2_R ModELAS device showing the blood and gas flow pathways (**a**). All three respiratory assist applications utilize an identical pumping compartment. Cannulation and HFM bundle size differentiate each respiratory assist configuration. Photograph of a study animal wearing the holstered device (**b**)

The adult low-flow ECCO_2_R ModELAS utilizes a 0.65-m^2^ cylindrical, stacked, uncoated polymethylpentene HFM bundle (Fig. 1a). The benchtop results of our previously reported ECCO_2_R device utilized an identical bundle [17]. The primary difference between the previous and current version of the device are washout holes within the impeller. The targeted CO_2_ removal for this device is at least 35% of the metabolically produced CO_2_ in humans (~ 70 mL/min at normocapnia and at rest) at a blood flow of 500 mL/min.

The adult low-flow ECCO_2_R ModELAS was evaluated for 7 days in healthy, 41.2–54 kg Suffolk sheep (*n* = 6; 5 males, 1 female; 7–12 months of age) at the McGowan Institute for Regenerative Medicine’s Center for Preclinical Studies (CPCS). The animal care and surgical protocol was approved by the University of Pittsburgh’s Institutional Animal Care and Use Committee (Protocol# 17101264). All animals had unrestricted access to food and water and were able to stand and lie down. CPCS staff assessed the animals’ well-being, habitus, and pain regularly. Prior to the study, the animals were conditioned to the fixed-point tethering system and the modified backpack which held the device during the study.

The animals were fasted at least 12 h prior to the surgery. Anesthesia was induced via a subcutaneous injection of atropine (0.25 mg/kg) followed by intravenous administration of ketamine (4 mg/kg) and midazolam (0.4 mg/kg). The animals were then directly intubated using a cuffed McGill-type endotracheal tube and anesthesia was maintained with isoflurane.

Venous and arterial pressure lines were placed in the left external jugular vein and carotid artery, respectively. These lines were secured at the vessel, tunneled underneath the skin, and left in place for the entirety of the study. The right external jugular vein was exposed via a surgical cut down. The target pre-catheterization activated clotting time (ACT) was above 300 s (ACT-II, Medtronic, Minneapolis, MN) and was achieved with a 180 IU/kg heparin bolus. Additional 1000–2000 IU heparin boluses were administered to increase the ACT above 300 s if necessary. The right external jugular vein was cannulated with a 15.5 Fr dual-lumen catheter (ALung Technologies, Pittsburgh, PA). The catheter was advanced to the right atrial-superior vena cava junction. The catheter position was confirmed via fluoroscopy. The catheter was secured to the neck via sutures and further protected with a neck wrap.

The primed adult low-flow ECCO_2_R ModELAS (4 IU/mL heparinized normal saline) was connected to the catheter, and extracorporeal blood flow was initiated at 0.5 L/min. Pure oxygen sweep gas was initially set to 4 L/min. The animal was recovered from anesthesia in the CPCS intensive care unit and monitored 24 h a day. The animal wore the device in a modified backpack (Fig. 1b). The animal was tethered within a pen and able to freely stand, lay, and move its head during the study. Prophylactic antibiotics were given every 8 h (25 mg/kg cephazolin) and an analgesic (1 mg/kg flunixin meglumine) was administered as needed. Mean arterial pressure (MAP), central venous pressure (CVP), and heart rate were recorded hourly. ACT was measured at least every 8 h post-operatively and more frequently when heparin titration was required.

Prior to euthanasia, a heparin bolus (15,000 IU) was administered to increase the ACT to greater than 300 s. After confirmation of the ACT, the ModELAS pump was turned off and the device removed from the animal, gravity flushed with saline, and examined for thrombus. SomnaSol (*pentobarbital sodium*, Henry Schein Animal Health, Dublin, OH) was then administered, and cessation of the heartbeat was confirmed by the CPCS veterinarian.

CO_2_ removal rate (${\dot{\mathrm{V}}}_{{\mathrm{CO}}_2}$) was measured daily and is reported as the average of 10 min of data collected at a sampling frequency of 2 Hz. Reported ${\dot{\mathrm{V}}}_{{\mathrm{CO}}_2}$ was collected at a sufficiently high sweep gas flow rate (Q_SG_) that the ${\dot{\mathrm{V}}}_{{\mathrm{CO}}_2}$ was not a function of sweep gas flow rate [18]. The ${\dot{\mathrm{V}}}_{{\mathrm{CO}}_2}$ was measured as the fraction of CO_2_ in the outlet sweep gas (${\mathrm{F}}_{{\mathrm{CO}}_2}$) (WMA-4 Analyzer, PP Systems, Amesbury, MA). The ${\dot{\mathrm{V}}}_{{\mathrm{CO}}_2}$ was calculated according to Eq. 1 and normalized to an inlet pCO_2_ of 45 mmHg according to Eq. 2 in order to reduce variability resulting from fluctuations of the inlet pCO_2_ [17]:

1 $$ {\dot{\mathrm{V}}}_{{\mathrm{CO}}_2}={\mathrm{Q}}_{\mathrm{SG}}{\mathrm{F}}_{{\mathrm{CO}}_2} $$

2 $$ {\dot{\mathrm{V}}}_{{\mathrm{CO}}_2}^{\ast }={\dot{\mathrm{V}}}_{{\mathrm{CO}}_2}\frac{45\mathrm{mmHg}}{{\mathrm{P}}_{{\mathrm{CO}}_2}^{\mathrm{Inlet}}} $$

The inlet pCO_2_ (${\mathrm{P}}_{{\mathrm{CO}}_2}^{\mathrm{Inlet}}\Big)$ used to normalize the ${\dot{\mathrm{V}}}_{{\mathrm{CO}}_2}$ is the average of the device inlet pCO_2_ taken immediately before and after data collection (${\mathrm{P}}_{{\mathrm{CO}}_2}^{\mathrm{Inlet}}$). During periods of low HCT (below 18%), device inlet pCO_2_ was only taken prior to data collection.

Plasma-free hemoglobin (PfHb), blood chemistry, and complete blood count (CBC) were measured pre-operatively and post-operatively on pre-operative days 6 and 1 and on POD (post-operative day) 0, 1, 3, 6, and 7. The blood sampling schedule was modified if the HCT was below 18% in order to reduce the amount of blood drawn from the animal. The reported pre-operative measurements are an average of data from both pre-operative days. POD 0 data is an average of samples taken when the arterial line was placed, initiation of extracorporeal blood flow and 4 h post-operatively. PfHb and platelet activation were measured using previously described methods [19–21]. Blood chemistry, manual platelet count, and CBC were measured by an external lab (IDEXX Laboratories, Inc., Westbrook, ME). Liver and kidney function and tissue injury were monitored by aspartate transferase (AST), alanine transferase (ALT), alkaline phosphatase (ALP), blood urea nitrogen (BUN), creatinine, and creatine kinase (CK). Platelet activation was measured via flow cytometry as previously reported [20, 21]. Platelet function was quantified by activating platelets with platelet activating factor (PAF).

Blood chemistry, platelet count, and CBC samples were not drawn on POD 6 for animals 3 and 6 due to low hematocrit. The blood chemistry for animal 4, pre-operative day 6 and manual platelet count for animal 6, preoperative day 1 and POD 1 and 3 were not measured due to a technical oversight.

## Statistical analysis

Data points were averaged across studies for each POD and are reported as the average ± standard deviation. Statistical analysis was completed using IBM SPSS (IBM Corporation, North Castle, NY). Due to the instances of missing data previously described, the statistical analysis was completed using a mixed linear model analysis of variance and the restricted maximum likelihood estimation method [22]. Raw extracorporeal blood flow rate, motor torque, heart rate, CD62P expression, creatinine, CK, ALT, and BUN data violated the assumption of normality based on the Kolmogorov–Smirnov test and were normalized [23] prior to statistical evaluation. For those comparisons in which time had a significant effect, pairwise comparisons were conducted using least significant difference analysis. Statistical significance was considered at *p* < 0.05.

## Results

All animals were successfully recovered from the surgery, and five studies were electively terminated on POD 7. Animal 4 was terminated early on POD 4 due to low MAP and an elevated lactate level. The necropsy showed significant pulmonary emboli within both lungs. Histopathological examination of the pulmonary emboli demonstrated that the initial emboli likely occurred several days prior to the end of study. On POD 1 of animal 4, the extracorporeal blood flow rate was 0.0 L/min for approximately 1 h. Blood pressure within the device was elevated, indicating an occlusion post-device. The device inflow tubing was clamped off while saline was forced through the device outflow tubing, and this returned blood flow to 0.5 L/min. Significant anemia was observed during animal 3 due to a non-study-related hematoma within the right scapular/axillary region. No thrombus or free fluid was evident within the chest or pericardium; thus, the scapular hematoma was likely due to accidental trauma to the shoulder unrelated to the device. Animal 5 was terminated approximately 3 h prior to the planned end of study due to a fractured positive pressure port on the device used to measure bundle inlet pressure on the prototype device. Significant blood loss through the fractured port necessitated rapid euthanasia, and POD 7 blood samples for blood chemistry, CBC, and gas exchange data were not collected.

**Fig. 2:**
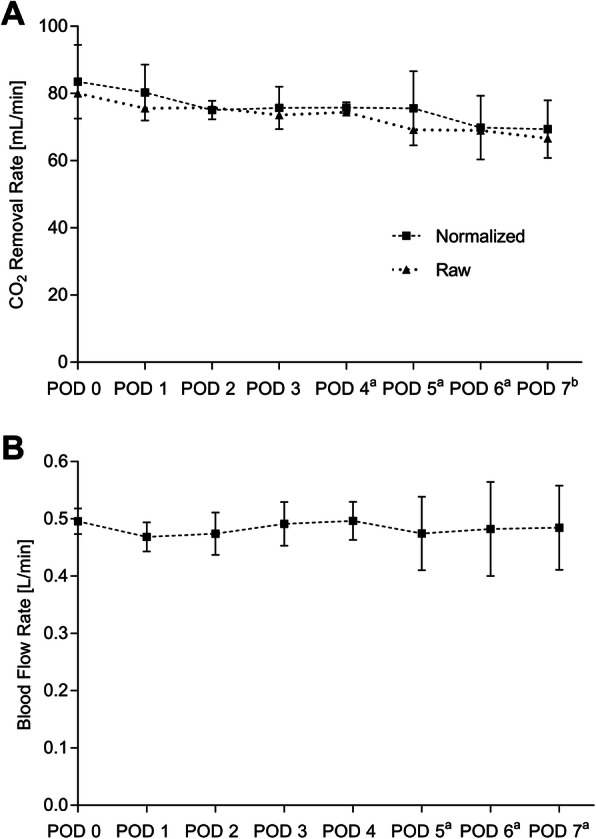
Raw and normalized CO_2_ removal rates (**a**) and daily extracorporeal blood flow rate (**b**). When compared to the POD 0 data, neither parameter significantly changed over duration of the study (*p* > 0.05). ^a^*n* = 5, ^b^*n* = 4

Figure 2 provides average extracorporeal blood flow rate and ${\dot{\mathrm{V}}}_{{\mathrm{CO}}_2}$. Average blood flow rate across all animals was 0.48 ± 0.01 L/min and did not vary significantly over the course of the 7 days (*p* = 0.338). Average CO_2_ removal (normalized to an inlet of 45 mmHg) across all animals and time points was 75.6 ± 4.7 mL/min. Daily average ${\dot{\mathrm{V}}}_{{\mathrm{CO}}_2}$ did not vary significantly from POD 0 (*p* = 0.233). The average device inlet pCO_2_ was 43.6 ± 1.1 mmHg. The device inlet pCO_2_ values measured before and after the ${\dot{\mathrm{V}}}_{{\mathrm{CO}}_2}$ data collection did not deviate by greater than 15%.

**Fig. 3:**
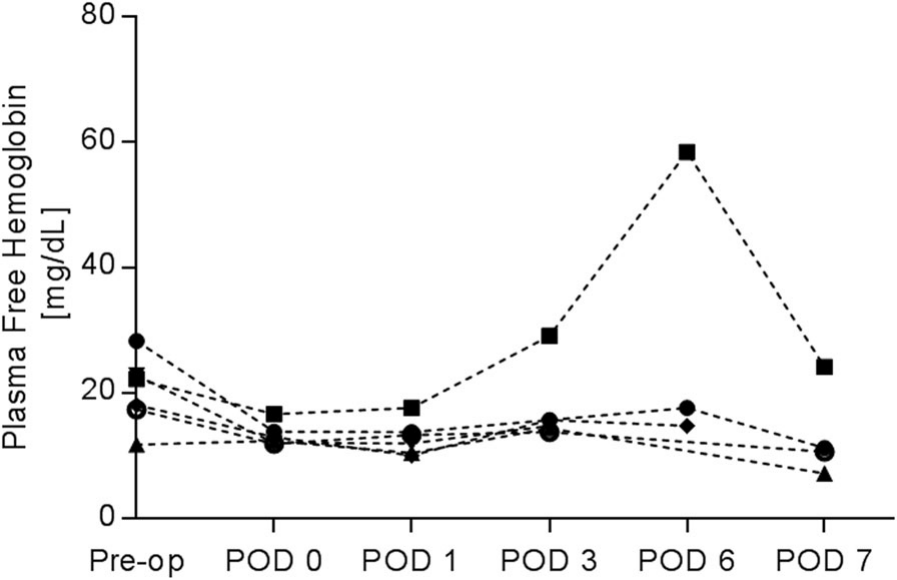
Plasma-free hemoglobin generated during the adult low-flow ECCO_2_R ModELAS in vivo study. Legend: ● animal 1; ■ animal 2; ▲ animal 3; ▼ animal trial 4; ♦ animal 5; ○ animal 6

MAP, CVP, and heart rate did not significantly change from POD 0 (Table 1). The negative CVP was likely due to the pressure monitoring line’s proximity to the 15.5 Fr DLC catheter drainage holes. Table 2 provides the pre-operative and post-operative animal hematologic parameters used to evaluate end organ function and hemocompatibility. Platelet function and activation did not statistically change from pre-operative values. BUN statistically increased on POD 6 prior to returning to pre-operative values on POD 7. HCT post-operatively was statistically less than pre-operative measurements. Post-operative pfHb remained below 25 mg/dL with the exception of POD 3–5 of animal 2 prior to returning to a normal level on POD 7 (Fig. 3). This animal showed signs of an acute kidney injury on POD 4 which resolved on POD 6. Macroscopic and microscopic examination showed no abnormalities or damage to the heart, lungs, liver, kidneys, or spleen, except animal 4 which exhibited signs of a pulmonary embolism.

**Table 1:** Hemodynamic and device parameters

Parameter	POD 0	POD 1	POD 2	POD 3	POD 4	POD 5^***a***^	POD 6^***a***^	POD 7^***a***^
Mean arterial pressure [mmHg]	91 ± 5	91 ± 7	89 ± 7	93 ± 5	96 ± 8	104 ± 5^*b*^	100 ± 7^*b*^	96 ± 7
Central venous pressure [mmHg]	− 1 ± 2	− 2 ±2	− 2 ± 2	− 2 ± 1	− 1 ± 2	− 3 ± 2	− 1 ± 3	− 2 ± 2
Heart rate [BPM]	94 ± 2	85 ± 13	92 ± 23	107 ± 25	124 ± 31^*b*^	111 ± 19	119 ± 27	119 ± 44
Speed [RPM]	1208 ± 80	1212 ± 47	1223 ± 54	1231 ± 53	1225 ± 54	1228 ± 59	1227 ± 62	1227 ± 60
Torque [mN-m]	10 ± 1	10 ± 1	10 ± 1	10 ± 1	10 ± 1	10 ± 2	10 ± 2	11 ± 3

^*a*^Animal 4 data not collected due to early termination

^*b*^Statistically significant compared to POD 0 (*p* < 0.05)

**Table 2:** Hematologic and end organ function parameters

Parameter	Pre-Op	POD 0	POD 1^*a*^	POD 3^*b*^	POD 6^*c*^	POD 7^*d*,*e*^
PAF activated [%]	71 ± 9	70 ± 10	77 ± 11	72 ± 11	75 ± 12	76 ± 3
CD62P [%]	3 ± 1	4 ± 2	2 ± 1	5 ± 5	5 ± 3	4 ± 2
Platelet count [k/μL]	576 ± 252	397 ± 110	499 ± 186	703 ± 281	520 ± 115	552 ± 198
WBC [K/μL]	9 ± 3	7 ± 2	12 ± 4	11 ± 4	12 ± 5	12 ± 1.7^*f*^
HCT [%]	36 ± 3	24 ± 2^*f*^	29 ± 2^*f*^	27 ± 6^*f*^	24 ± 4^*f*^	27 ± 7^*f*^
Creatinine [mg/dL]	1 ± 0	1 ± 0	2 ± 1	3 ± 3	1 ± 0	1 ± 0
BUN [mg/dL]	11 ± 2	11 ± 3	11 ± 5	27 ± 21	26 ± 16^*f*^	14 ± 4
CK [U/L]	103 ± 23	155 ± 51	143 ± 72	71 ± 11	149 ± 88	126 ± 80
ALT [U/L]	12 ± 5	9 ± 3	9 ± 4	5 ± 3	5 ± 2	5 ± 5
AST [U/L]	71 ± 14	58 ± 11	73 ± 19	56 ± 10	53 ± 12	59 ± 9
ALP [U/L]	218 ± 56	165 ± 55	158 ± 41	141 ± 33	101 ± 26	128 ± 64

*ALT* alanine transferase, *ALP* alkaline phosphatase, *AST* aspartate aminotransferase, *BUN* blood urea nitrogen, *CK* creatine kinase, *HCT* hematocrit, *PAF* platelet-activating factor, *WBC* white blood cell count

^*a*^Missing platelet count from animal 6

^*b*^Missing platelet count from animals 2, 5, and 6

^*c*^Missing all data, except HCT, for animals 3, 4, and 6

^*d*^Missing all data, except HCT, for animals 4 and 5

^*e*^Missing CD62P and PAF activated for animal 3

^*f*^Statistically significant compared to pre-op value (*p* < 0.05)

**Fig. 4:**
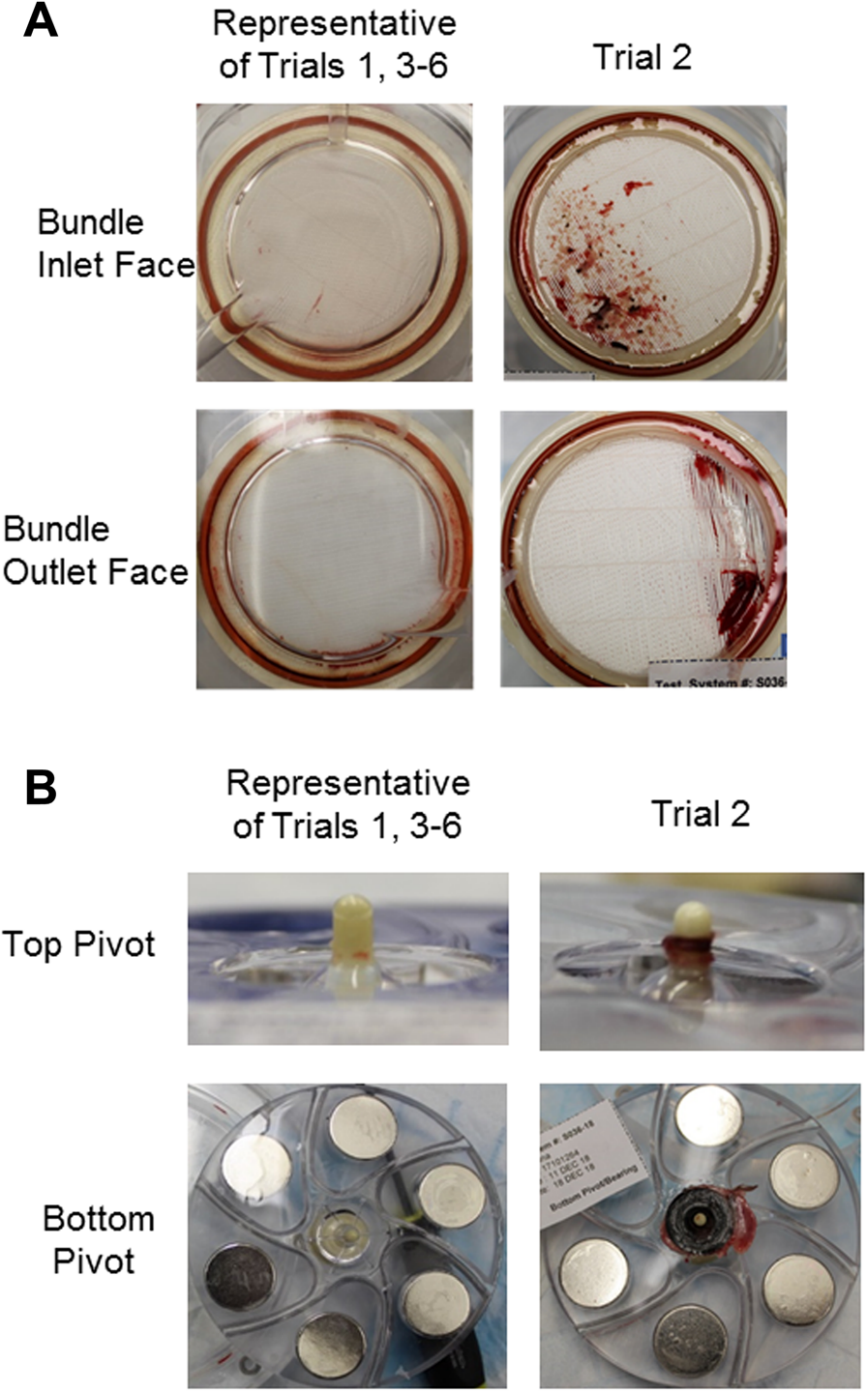
(**a**) Images of the explanted hollow fiber membrane bundle inlet (*top row*) and outlet (*bottom row*) faces and the (**b**) impeller top (*top row*) and bottom (*bottom row*) pivots. The animal 2 (*right*) bundle and pivots had significant thrombus formation due to low ACT. All other animals were free of significant thrombi (*left*)

The explanted device from animal 2 had thrombus at the bottom pivot of the impeller and within the bundle (Fig. 4). During this animal study, the ACTs were below the target range for a quarter of the study. All other explanted devices were free of significant thrombus.

## Discussion

The Modular Extracorporeal Lung Assist System (ModELAS) is an integrated, modular pump-lung capable of providing three levels of respiratory assistance. Selection of the cannula and hollow fiber membrane bundle configures the ModELAS for pediatric complete respiratory assist, adult complete respiratory assist, or adult low-flow CO_2_ removal. The present study validated benchtop gas exchange performance and evaluated the thrombogenicity and hemocompatibility of the adult low-flow ECCO_2_R ModELAS in a healthy ovine model for 7 days. All animals were successfully recovered from the surgery, and a single animal required early termination on POD 4. No device exchanges were required.

These preclinical studies indicate that the adult low-flow ECCO_2_R ModELAS can operate for 7 days without diminished CO_2_ removal performance nor significant damage to blood or end organs. The average ${\dot{\mathrm{V}}}_{{\mathrm{CO}}_2}$ was 75.6 mL/min, and the ${\dot{\mathrm{V}}}_{{\mathrm{CO}}_2}$ on POD 7 was 83% of that on POD 0. Low HCT resulting from a shoulder hematoma (animal 3), pulmonary embolism (animal 4), and subcutaneous bleeding (animal 5) resulted in slightly diminished CO_2_ removal rates [24, 25] and increased blood flow rates during the latter half of the study. The in vivo CO_2_ removal performance on POD 7 was within 5% of previously published benchtop ModELAS ${\dot{\mathrm{V}}}_{{\mathrm{CO}}_2}$ data [17, 25], hence validating benchtop performance.

The adult low-flow ECCO_2_R ModELAS was generally well tolerated by the animals. Platelet and organ function parameters used to evaluate device safety did not significantly change during the study (Table 2). Except animal 4, macroscopic and histopathological evaluation of the end organs did not indicate any device-related pathology. Pre-operative pfHb samples were drawn via a venous puncture and are therefore slightly elevated. A single study (animal 2) had elevated pfHb measurements greater than the 40–50 mg/dL clinically relevant threshold [26–28]. The maximum pfHb value occurred on POD 6 before decreasing on POD 7. The elevated pfHb was not associated with any hematuria. Animal 2 was also the sole animal in which the explanted device contained significant thrombus at the pivot bearing, likely caused by periods of improperly concentrated heparin infusion solutions. The thrombus and subsequent friction at the pivot bearing are possible contributors to the elevated pfHb. In addition, just prior to this period of elevated pfHb, the animal displayed symptoms of an acute kidney injury. The combination of the pivot thrombus and the kidney injury likely caused the sharp increase in pfHb in animal 2. Elevated pfHb was isolated to one out of the six studies, rather than a trend, and is not indicative of significant safety concerns.

The most significant device-related adverse event was a pulmonary embolism during animal 4. This animal required early termination on POD 4 due to low MAP and elevated heart rate and blood lactate concentration. The root cause of these symptoms was an acute pulmonary embolism in both lungs. This thrombus initiated several days prior to the end of study based on histopathological examination of the thrombus organization. Thromboembolism is a known risk of connecting any device to the vasculature including current artificial lungs [29, 30] and ventricular assist devices [31, 32]. In preclinical testing, the key is to differentiate whether the thromboembolism is inherent to the device design or was independent of the device used. The densely packed hollow fiber membrane bundle of the ModELAS acts as a filter; hence, it is unlikely that the thrombus was generated within the pump and migrated post-device (Fig. 1). Thus, the precipitating thrombus likely originated between the outlet of the device and the cannula tip, possibly at the tubing connectors [33–35], and was dislodged on POD 1 with the saline flush. The exact cause of the original thrombus remains unknown; however, it was an isolated event and does not appear to be characteristic of the device. The entire ModELAS circuit is also uncoated, whereas most commercial artificial lung circuits are coated with a biocompatible coating [36, 37]. Our laboratory is currently developing a tip-to-tip zwitterionic surface coating which will further improve hemocompatibility and further reduce thrombus formation within the ModELAS device, tubing, and catheter [38].

The most relevant comparison to the adult low-flow ModELAS is the Hemolung RAS (ALung Technologies, Pittsburgh, PA) which is currently undergoing an FDA clinical trial (VENT-AVIOD, NCT03255057) to evaluate the use of the Hemolung RAS in ae-COPD patients and the REST trial in the UK to assess the use of VV-ECCO_2_R in conjunction with the lung-protective ventilation in ARDS patients. In previously published pre-clinical healthy ovine studies, the Hemolung (0.59 m^2^) achieved 42–53 mL/min of CO_2_ removal at blood flow rates between 350 and 450 mL/min [27]. In comparison, the adult low-flow ECCO_2_R ModELAS CO_2_ removal rate ranged from 69 to 84 mL/min at blood flow rates ranging between 430 and 590 mL/min in the present study. The improved ${\dot{\mathrm{V}}}_{{\mathrm{CO}}_2}$ in the adult low-flow ECCO_2_R ModELAS could be a result of slightly higher blood flow rates and bundle surface area (0.65 m^2^); however, the ModELAS has the additional benefits of being designed as a wearable unit and being capable of a wider range of respiratory therapies.

## Conclusions

The ModELAS is a novel platform respiratory assist device. This preclinical study demonstrated that the low-flow ECCO_2_R ModELAS is capable of removing clinically significant amounts of CO_2_ at a blood flow rate of 500 mL/min consistently over 7 days in a healthy ovine model. The explanted devices were generally free of thrombus and only isolated device-related events occurred demonstrating positive hematologic and thrombotic compatibility of the ModELAS. The ModELAS is a promising platform respiratory assist technology, and further testing and product development is warranted.

## Data Availability

The datasets used and/or analyzed during the current study are available from the corresponding author on reasonable request.

## Abbreviations

ACT: Activated clotting time
ALT: Alanine transferase
ALP: Alkaline phosphatase
AST: Aspartate aminotransferase
ARDS: Acute respiratory distress syndrome
ae-COPD: Acute exacerbations of chronic obstructive pulmonary disease
AST: Aspartate transferase
BUN: Blood urea nitrogen
CBC: Complete blood count
CK: Creatine kinase
CVP: Central venous pressure
ECCO_2_R: Extracorporeal CO_2_ removal
${\mathrm{F}}_{{\mathrm{CO}}_2}$: Fraction of CO_2_ in the sweep gas
HCT: Hematocrit
MAP: Mean arterial pressure
ModELAS: Modular Extracorporeal Lung Assist System
MV: Mechanical ventilation
NIV: Noninvasive ventilation
PAF: Platelet-activating factor
PAP: Pulmonary arterial pressure
PfHb: Plasma-free hemoglobin
POD: Post-operative day
Q_SG_: Sweep gas flow rate
${\dot{\mathrm{V}}}_{{\mathrm{CO}}_2}$: CO_2_ removal rate
VILI: Ventilator-induced lung injury
WBC: White blood cell count
